# A Conserved Carboxylesterase Inhibits *Tobacco mosaic virus* (TMV) Accumulation in *Nicotiana benthamiana* Plants

**DOI:** 10.3390/v12020195

**Published:** 2020-02-10

**Authors:** Song Guo, Sek-Man Wong

**Affiliations:** 1 Department of Biological Sciences, National University of Singapore, Singapore 117543, Singapore; dbsguos@nus.edu.sg; 2 National University of Singapore (Suzhou) Research Institute, Suzhou 215123, China; 3 Temasek Life Sciences Laboratory, 1 Research Link, Singapore 117604, Singapore

**Keywords:** Carboxylesterase, *Tobacco mosaic virus*, host resistance, coat protein

## Abstract

A carboxylesterase (CXE) or carboxylic-ester hydrolase is an enzyme that catalyzes carboxylic ester and water into alcohol and carboxylate. In plants, CXEs have been implicated in defense, development, and secondary metabolism. We discovered a new CXE gene in *Nicotiana benthamiana* that is related to virus resistance. The transcriptional level of NbCXE expression was significantly increased after *Tobacco mosaic virus* (TMV) infection. Transient over-expression of NbCXE inhibited TMV accumulation in *N. benthamiana* plants. Conversely, when the NbCXE gene was silenced with a *Tobacco rattle virus* (TRV)-based gene silencing system, TMV RNA accumulation was increased in NbCXE-silenced plants after infection. NbCXE protein was shown to interact with TMV coat protein (CP) in vitro. Additionally, the expressions of host defense-related genes were increased in transient NbCXE-overexpressed plants but decreased in NbCXE silenced *N. benthamiana* plants. In summary, our study showed that NbCXE is a novel resistance-related gene involved in host defense responses against TMV infection.

## 1. Introduction

Carboxylesterases (CXEs) are found in all kingdoms of life. They are enzymes that catalyze the hydrolysis of carboxylic esters into their corresponding alcohols and carboxylic acids [1]. In animals and microbes, many CXEs have been cloned and characterized [2,3,4]. They are involved in a broad range of functions, including processing and degradation of neurotransmitters, hormones, and xenobiotics [5,6].

Carboxylesterases (CXEs) are also widely distributed in plants. They are expressed in many tissues, including fruits, leaves, and roots [7,8,9]. Plant CXEs have been implicated in defense, development, and secondary metabolism [10,11,12,13]. Some plant CXEs have developed new activities. For example, at least two CXEs in legumes have attained additional dehydratase activity and are involved in isoflavone biosynthesis [14]. In rice, a putative CXE has lost carboxylesterase activity and functions as a receptor for gibberellic acid [15].

Plant CXEs are classified into three classes based on sequence features. Classes I and II belong to the α/β hydrolase fold superfamily. Enzymes in class I share several sequence features, typically the His-Gly-Gly (HGG) motif and the Ser-His-Asp catalytic triad [16]. The first plant carboxylesterase to be identified was the *Hsr203J* protein of *N. tabacum*, which is induced during hypersensitive reactions (HRs) and possesses ester-hydrolyzing activity [17]. Enzymes in class II were originally identified as being related to flavin adenine dinucleotide (FAD)-independent hydroxynitrile lyases (HNLs) of cassava (*Manihot esculenta*) and the tropical rubber tree (*Hevea brasiliensis*) [18,19]. They have been shown to preferentially catalyze methyl ester hydrolysis. Class III enzymes are completely unrelated to α/β hydrolases and belong to the Gly-Asp-Ser (GDS) hydrolase family [16].

In most cases, a sequence annotated as “carboxylesterases” in the database means it belongs to the class I enzymes. For example, a family of 20 plant carboxylesterases have been identified from the *Arobidopsis thaliana* genome and designated as AtCXE1–AtCXE20. These AtCXEs genes are widely distributed across the genome, present in four out of five chromosomes. Multiple-sequence alignment results show conserved motifs among all AtCXEs [20].

Carboxylesterases are also a class of the metabolic enzymes that have been shown to be involved in the resistance of insects to organophosphate insecticides and pyrethroids through gene amplification, upregulation, and coding sequencing mutations [21]. CXEs also play important roles in herbicide activation. These plant CXEs detoxify persistent pollutants and insecticides, as well as hydrolyzing pro-herbicide esters to their bioactive free acids. As many major herbicides are applied as esters to facilitate penetration into the leaf, ester hydrolysis by plant CXEs is required to activate those herbicides [22].

Plants have evolved several mechanisms to protect themselves against pathogen attacks. Hypersensitive response is one of the most common and efficient plant reactions to pathogens [23]. The HR is characterized by rapid cell death in the local region surrounding an infection. The HR restricts the growth and spread of pathogens to other plant parts [24]. Hypersensitive cell death occurs via the recognition of pathogen avirulence (avr) genes by plant resistance (R) genes [25]. Besides direct R genes, there are also several hypersensitive-related genes that can trigger plant cell death. For example, *Hsr203J* is a tobacco gene associated with an HR to the bacterium *Ralstonia solanacearum* [26]. Also, the *Lehsr203* gene is another HR-related gene in tomato [27]. *SOBER1* in *Arabidopsis* functions as a negative regulator of the HR, triggered by the bacterial type III effector protein AvrBsT. Furthermore, *SOBER1* is not restricted to *A. thaliana* and only limited to suppress AvrBsT-induced HR [28].

*Chenopodium quinoa* and *C. amaranticolor* are local lesion hosts of many plant viruses. Using *Tobacco mosaic virus* (TMV)-tagged with green fluorescent protein (GFP) to infect *C. quinoa* and *C. amaranticolor*, a group of short fragment sequences were highly expressed in these two plants at 4 days post-TMV-GFP infection. Those short sequences in *C. amaranticolor* are termed as disease-expressed sequences in *C. ameranticolor* (DESCA) genes, which are closely associated with pathogen defense in plants. Expression of these DESCA genes are also induced by *Tobacco rattle virus* (TRV) infection. In *C. quinoa*, DESCA5 was increased 1,100 fold at 4 dpi after TMV infection [29]. The BLAST results showed that DESCA5 is similar to a yeast potential transcriptional regulator. The reason whyDESCA5 highly increased after TMV infection in *C. quinoa* is unknown.

In this study, we aligned the DESCA5 sequence to the whole genome of *C. quinoa* and found that it matched a carboxylesterase (CXE) gene. A similar gene in *N. benthamiana* was amplified and named NbCXE. Similar to that of DESCA5 in *C. quinoa*, the transcriptional level of NbCXE was also highly expressed in *N. benthamiana* at 4 dpi after TMV infection. Transient over-expression of NbCXE could inhibit TMV RNA accumulation. Conversely, silencing of NbCXE increased accumulation of TMV RNA and coat protein (CP) in infected leaves. Moreover, NbCXE could interact with TMV CP in a yeast two-hybrid system. Our study revealed that NbCXE is a newly discovered resistance-related gene in *N. benthamiana* and its expression inhibits TMV accumulation.

## 2. Materials and Methods

### 2.1. Plant Materials and Virus Inoculation

*N. benthamiana* plants were grown at 24 °C in a growth room under a 16 h light/8 h dark cycle. Fully expanded leaves of 4-week-old plants were mechanically inoculated with 2 µg in vitro transcribed viral RNA in a GKP buffer (50 mM glycine; 30 mM K_2_HPO_4_, pH 9.2; 1% bentonite; 1% celite).

### 2.2. Construction of NbCXE Over-Expression and TRV Silencing Vectors

The complete open reading frame (ORF) of the NbCXE gene was inserted into a pGreen vector with a GFP tag using primers F-NbCXE-*Eco*RI and R-NbCXE-*Bam*HI, termed 35S-NbCXE-GFP. The TRV binary vectors pTRV1 and TRV RNA2 were used for the silencing study. DNA fragments used for the silencing study were amplified from *N. benthamiana* cDNA by RT-PCR with primers F-NbCXE-*Eco*RI and R-NbCXE_400_-*Bam*HI. The nucleotide sequences of all the primers used are listed in Supplementary Table S1.

### 2.3. Transient Over-Expression of NbCXE and TRV-Based Gene Silencing of NbCXE

The vector 35S-NbCXE-GFP and TRV2:NbCXE silencing vectors were introduced into the *Agrobacterium* strain GV3101 by electroporation (BIO-RAD, Hercules, CA, USA) separately. *N. benthamiana* plants at the 6 to 10 leaf-stage were used for agroinfiltration. The appropriate binary plasmids in *Agrobacterium* were grown overnight at 28 °C and cultures were diluted to cell density of 0.6 at OD and 600 nm, and were infiltrated into the abaxial leaves of *N. benthamiana* immediately above the cotyledons using a 1 mL syringe.

For agroinfiltration-based transient over-expression of NbCXE, in vitro transcribed TMV RNA (2 µg) was inoculated in the infiltrated leave of plants at 3 days post-infiltration. The inoculated leaves were collected at 7 days post-inoculation for RNA isolation, Western blot analysis, and qRT-PCR experiments. For the TRV-induced NbCXE gene silencing system, in vitro transcribed TMV RNA (2 µg) was inoculated in the infiltrated leaves at 7 days post-infiltration. The inoculated leaves were collected at 7 days post-inoculation for further experiments.

### 2.4. Total RNA Isolation and Western Blot

For the NbCXE transient over-expression experiment, plant total RNAs were extracted from infiltrated leaves at 3 days post-infiltration to detect NbCXE transcriptional expression levels. For NbCXE silencing plants, the expression level of NbCXE was detected from infiltrated leaves and upper leaves at 7 days post-infiltration. Total RNA was isolated using TRIzol reagents, following the manufacturer’s instructions.

For both NbCXE transient over-expression and NbCXE-silenced experiments, three biological repeats were carried out in each treatment of the experiment. Each experiment was repeated two times. Total RNAs and total proteins were extracted from infected plants at 7 days post-inoculation. Total proteins were separated on 15% SDS-PAGE gel, then transferred to nitrocellulose membrane (Bio-Rad). The Western blot was carried out to detect TMV CP expression with anti-TMV CP antibody. The CP band was visualized using 5-bromo-4-chloro-3-indolyl phosphate and nitroblue tetrazolium (NBT/BCIP) from Sigma-Aldrich Chemical Company (St Louis, MO, USA). The band intensity was quantified using the ImageJ software (https://imagej.nih.gov/ij/).

### 2.5. qRT-PCR Analysis

The complete ORF of NbCXE was amplified from total RNA of healthy *N. benthamiana* plants through RT-PCR using the Superscript III^TM^ First-Strand Synthesis System (Invitrogen, Carlsbad, CA, USA) and KOD-plus-Neo DNA polymerase (Toyobo, Tokyo, Japan) with primers NbCXE-F and NbCXE-R. qRT-PCR was performed in triplicate with KAPA SYBR on the CFX384 Real-Time PCR system (Bio-Rad) with primers NbCXE-qF and NbCXE-qR. The *actin* gene was used as an internal control with primers *actin*-qF and *actin*-qR. The experiment was repeated twice. To quantify TMV RNA accumulation in inoculated plants, total RNA from each of the triplicate samples of each treatment was extracted and reverse transcribed by using Superscript III Reverse Transcriptase (Life Technologies, Invitrogen) with primer TMV-R. qRT-PCR was performed in triplicate with primers TMV-qF and TMV-qR, using the *actin* gene as an internal control.

To quantify fold changes of selected host response genes (*NPR1*, *HIN1*, *HSP203J*, *PR1*, and *PR3*) in transient NbCXE over-expressed and NbCXE-silenced *N. benthamiana*, total RNA from each of the triplicate samples of each treatment was extracted and reverse transcribed using Superscript III Reverse Transcriptase (Life Technologies, Invitrogen) with a specific reverse primer. qRT-PCR was performed in triplicate with specific forward and reverse primers (Supplementary Table S1). The *actin* gene was used as an internal control. Each experiment was repeated twice.

For qRT-PCR analysis, the 5 µL reaction mixture contained 20 ng of cDNA, 200 nM of each pair of target primers, and 2.5 µL of 2× SYBR Green PCR Master Mix. PCR conditions were as follows: 95 °C for 3 min, followed by 40 cycles of 95 °C for 10 s and 60 °C for 30 s. Three technical replicates from three independent biological replicates were performed for qRT-PCR. The data were analyzed according to the 2^−ΔΔ*C*T^ method. The *actin* gene was used as a reference gene for analysis. Each experiment was repeated twice.

### 2.6. Yeast Two-Hybrid Assay

The full-length TMV CP gene was PCR amplified and cloned into a yeast pGADT7 vector (BD Biosciences Clontech, Palo Alto, CA, USA) to generate AD-TMV CP using primers F-TMV CP-*Eco*RI and R-TMVCP-*Bam*HI. The full-length NbCXE gene was PCR amplified and cloned into the pGBKT7 vector (BD Biosciences Clontech) to generate BD-NbCXE with primers F-NbCXE-*Eco*RI and R-NbCXE-*Bam*HI. The yeast strain AH109 (BD Biosciences Clontech) was co-transformed with 500 ng of individual plasmids using the polyethylene glycol-lithium acetate (PEG/LiAC) method. The transformants were cultured on a synthetic drop-out (SD) medium containing all amino acids except leucine and tryptophan (SD-Leu-Trp) at 30 °C for 3–4 days. Empty vector AD with BD-NbCXE and empty vector BD with AD-TMVCP were performed as controls.

A single colony was selected from each of the transformants and grown in 2 mL of SD-Leu-Trp medium at 30 °C for 24 h. The culture densities were normalized with SD-Leu-Trp, followed by three 10-fold serial dilutions. Ten µL each of the 1:1000 diluted cultures were grown on SD-Leu-Trp and SD-Leu-Trp-His-Ade growth medium at 30 °C for 3–4 days. Amino acids Leu, Trp, His, Ade denote leucine, tryptophan, histidine, and adenine, respectively. The experiment was repeated twice.

## 3. Results

### 3.1. Identification of the New Resistance-Related Gene NbCXE in N. benthamiana Plants

The DESCA5 sequence was registered in the NCBI database as “DESCA5 cDNA-AFLP *Chenopodium amaranticolor* cDNA EcoRI, mRNA sequence” with a GenBank accession number of BI534451.1. After aligning the DESCA5 sequence against the whole genome of *C. quinoa*, it showed a 96.40% identity with the sequence of “Predicted: *C. quinoa* probable carboxylesterase (LOC110718167), mRNA”. As *N. benthamiana* is a model plant for virus study and a systemic host of TMV, we decided to investigate the CXE gene in relation to TMV infection in *N. benthamiana* plants.

**Figure 1:**
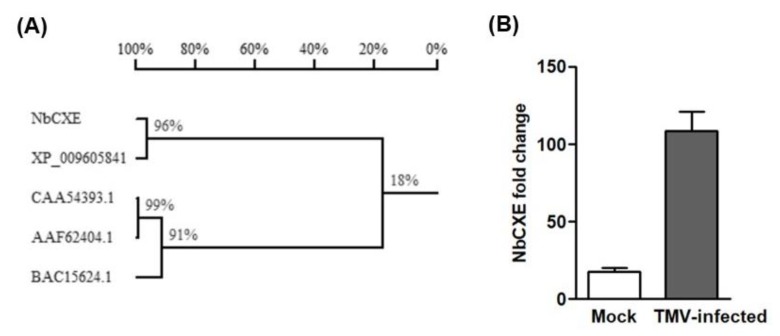
(**A**) Alignment of amino acid sequence of NbCXE with carboxylesterase in *Nicotiana sylvestris* and the *Hsr203J* proteins in *Nicotiana tabacum*. (XP_009605841 are the carboxylesterases in *Nicotiana sylvestris*; CAA54393.1, BAC15624.1 and AAF62404 are *Hsr203J* proteins in *Nicotiana tabacum*). (**B**) Expression of NbCXE showed a significant increase at 4 dpi in TMV-infected *N. benthamiana* plants.

Based on the *N. sylvestris* carboxylesterase (XP_009605841) sequence for primer design, the carboxylesterase gene of *N. benthamiana* (NbCXE) was amplified successfully. In order to ascertain whether this NbCXE is a plant resistance-related protein, amino acid sequences of the amplified NbCXE were compared with three known *Hsr203J* protein sequences in *N. tabacum* (AAF62404.1, BAC15624.1 and CAA54393.1). The amino acid sequences of NbCXE showed a low identity with the *Hsr203J* protein of *N. tabacum,* which indicated that it is not related to the *Hsr203J* resistance protein (Figure 1A). However, the expression of NbCXE showed a significant increase in expression at 4 dpi in TMV-infected plants (Figure 1B), similar to that of DESCA5 in *C. quinoa*, which suggested that it is related to resistance against TMV infection.

### 3.2. Over-Expression of NbCXE Inhibited TMV Accumulation in N. benthamiana Plants

**Figure 2:**
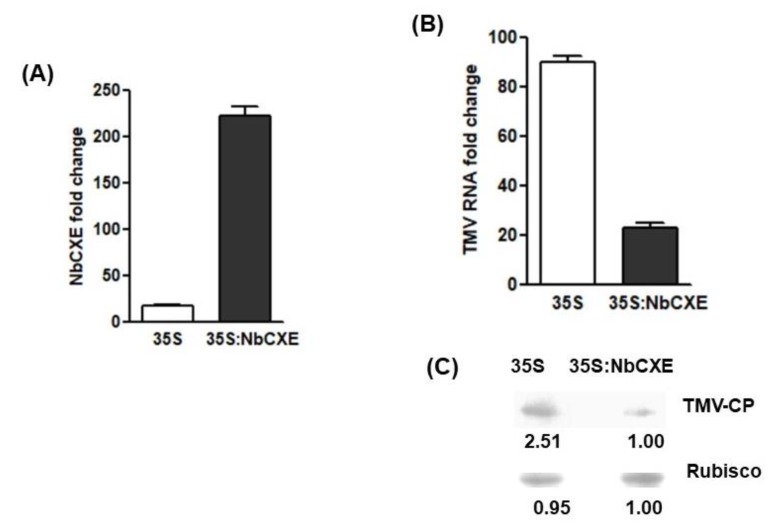
(**A**) After transient over-expression of NbCXE in *N. benthamiana* plants, the transcriptional level of NbCXE was increased at 3 days post-infiltration. (**B**) TMV RNA accumulation in the NbCXE-overexpressed leaves was significantly lower than that in the control plants. (**C**) NbCXE transient overexpressed leaves accumulated a lower level of TMV CP, as compared with that of the control plants.

Since *NbCXE* was upregulated after TMV infection, over-expression of NbCXE should inhibit TMV accumulation. To test this hypothesis, NbCXE was transiently over-expressed through agroinfiltration. The transcriptional level of NbCXE was increased at 3 days post-infiltration (Figure 2A). At 7 dpi, TMV RNA accumulation in the inoculated leaves of 35S NbCXE-infiltrated plants was significantly lower than that of the inoculated leaves of 35S vector-infiltrated control plants (Figure 2B). The NbCXE transiently over-expressed leaves accumulated a lower amount of TMV CP (Figure 2C). These results confirmed that the over-expression of NbCXE resulted in lower TMV accumulation in the infected plants.

### 3.3. Silencing of NbCXE Enhanced TMV Accumulation

**Figure 3:**
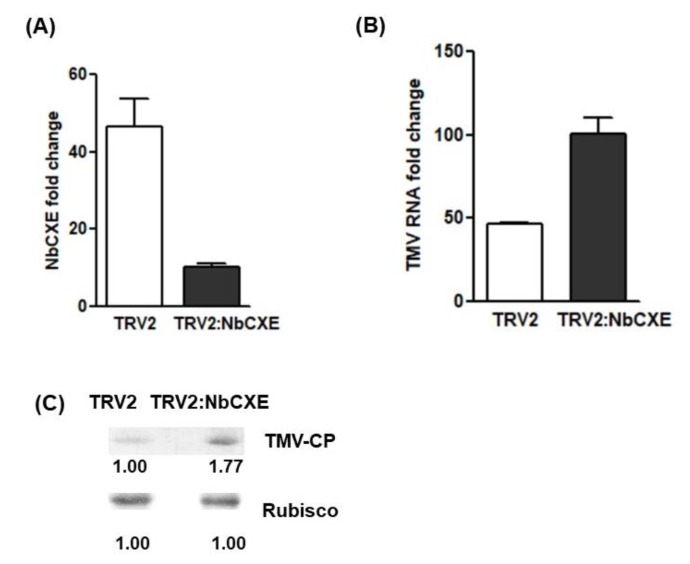
(**A**) The transcriptional level of NbCXE was down-regulated about 40 fold in the NbCXE-silencing plants, as compared to that of control plants. (**B**) Higher TMV RNA accumulation was detected in the NbCXE silencing plants than in the control plants. (**C**) Higher TMV CP was detected in the NbCXE silencing plants, as compared with that of control plants.

NbCXE was silenced through the TRV gene silencing system. After agroinfiltration, the transcriptional level of NbCXE was downregulated about 40 fold in the infiltrated leaves of plants (Figure 3A). Subsequently, TMV was inoculated to the infiltrated leaves and the accumulation of TMV RNA and CP after 7 dpi was monitored. The results showed that more TMV RNA (Figure 3B) and CP (Figure 3C) were accumulated in the NbCXE silencing plants than the control plants. Silencing of NbCXE enhanced higher TMV accumulation in plants.

### 3.4. Interaction of NbCXE with TMV CP

**Figure 4:**
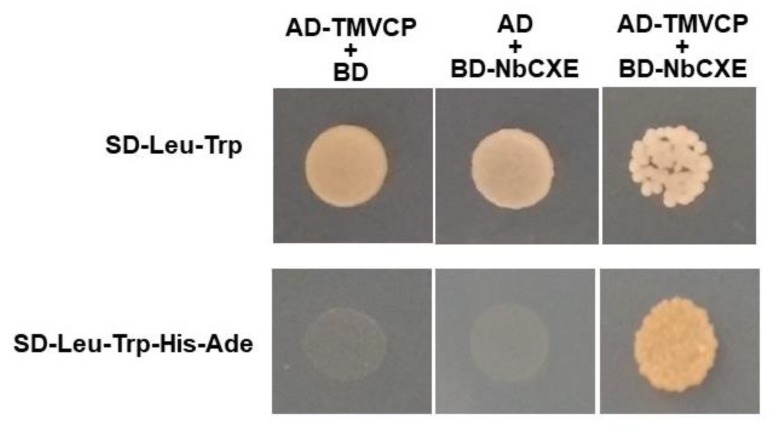
Yeast two-hybrid results showed an interaction between NbCXE and TMV CP.

TMV CP plays an important role in TMV replication. Since expression of NbCXE in plants affects TMV accumulation, there might be an interaction between NbCXE and TMV CP. A yeast two-hybrid experiment was performed and showed an interaction between NbCXE and TMV CP (Figure 4).

### 3.5. Expression of Host Defense-Related Genes Increased in Transient NbCXE-Overexpressed Plants but Decreased in NbCXE-Silenced N. benthamiana Plants

**Figure 5:**
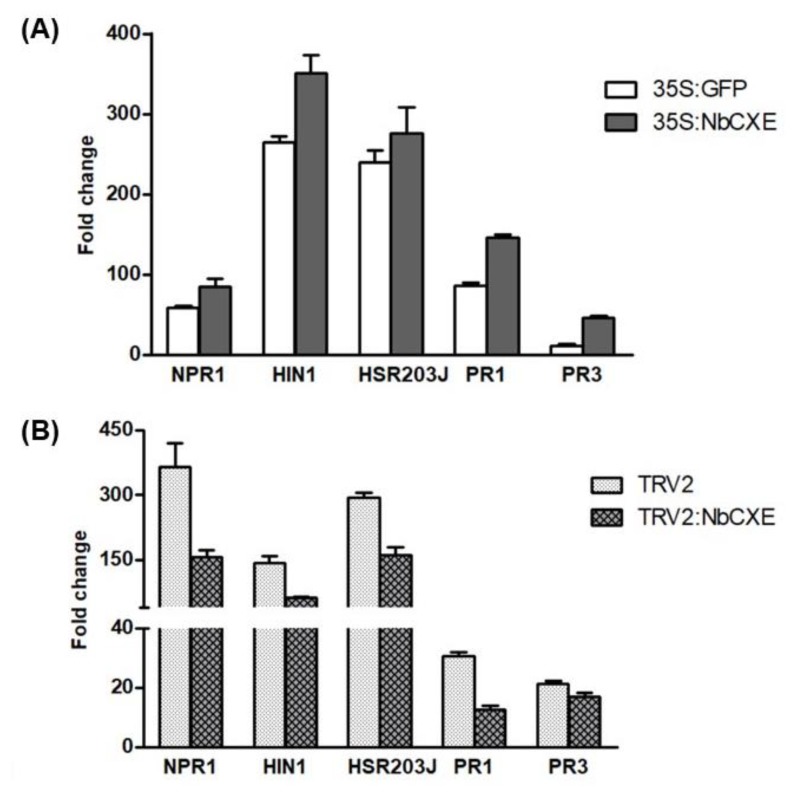
After TMV infection, the fold changes of selected host response genes (*NPR1*, *HIN1*, *HSP203J*, *PR1* and *PR3*) in (**A**) transient NbCXE-overexpressed and (**B**) NbCXE-silenced *N. benthamiana*, respectively.

To analyze the effects of NbCXE in host defense signaling, we examined the expression of several host defense-related genes in transient NbCXE-overexpressed plants and NbCXE-silenced plants after TMV infection, respectively. These genes included the important defense regulatory gene *NPR1*, HR marker genes *HIN1* and *Hsr203J*, and the two pathogenesis-related (PR) genes *PR1* and *PR3*. These host defense-related genes showed a higher expression in transient NbCXE-overexpressed plants but decreased in NbCXE-silenced plants (Figure 5A,B). This indicated that NbCXE is involved in host defense responses. Overexpression or silencing of NbCXE affects the expression levels of defense-related genes in host plants.

## 4. Discussion

### 4.1. NbCXE Encoded a Resistance-Related Protein

Resistance (R) genes in plant genomes convey plant disease resistance against pathogens by producing R proteins. The main class of R genes consists of a nucleotide binding domain (NB) and leucine-rich repeat (LRR) domains. The NB domain binds either ATP/ADP or GTP/GDP. The LRR domain is involved in protein-protein interactions as well as ligand binding [30,31]. Some R proteins can interact directly with a virulent protein produced by a pathogen [32,33,34]. They may detect a pathogen-associated molecular pattern or encodes enzymes that degrade toxins produced by pathogens [35]. Once the R protein has detected a pathogen, it will stimulate the host defense system against the pathogen [36]. TMV virulent protein is located in its helicase domain of the 126-kD replicase [37,38]. As NbCXE was increased after TMV infection and the expression of NbCXE in plants significantly inhibited TMV RNA accumulation after infection, it can be suggested that NbCXE is a plant resistance-related protein.

Carboxylesterase is an important class of detoxification enzymes involved in insecticide resistance. The relative transcriptional levels of carboxylesterases are much higher in resistant strains than in susceptible strains. Over-expression of carboxylesterase contributes to increased insecticide omethoate resistance in cotton aphids [39]. Oral delivery-mediated RNA silencing of the carboxylesterase gene in cotton aphids reduced its resistance to insecticides. This shows that insect CXE gene plays a major role in insecticide resistance [40]. Our results showed that the NbCXE gene is important for resistance against TMV infection in *N. benthamiana* plants. Over-expression of NbCXE showed an enhanced defense response to TMV infection, while silencing of NbCXE compromised host resistance to TMV infection and resulted in an increased viral RNA accumulation in infected plants.

Transgenic pepper plants overexpressing a pepper carboxylesterase gene exhibit a higher resistance against multiple fungal pathogens [41]. Moreover, applying the pepper carboxylesterase protein on the surface of unripe pepper fruits leads to disease resistance in the fruits, including generation of hydrogen peroxide and expression of pathogenesis-related (PR) proteins with antimicrobial activity [41]. In our study, the transiently over-expressed NbCXE enhanced plant resistance against TMV infection. The important defense regulatory gene *NPR1* and HR marker genes *HIN1* and *Hsr203J*, *PR1*, and *PR3* were all upregulated in transient NbCXE-overexpressed plants, implying an enhanced host defense response to TMV infection. Conversely, these host defense-related genes were reduced in expression in NbCXE-silenced plants, indicating that the host defense resulted in a higher TMV RNA accumulation in host plants. All these results indicated that NbCXE is a resistance-related gene associated with host defense response.

### 4.2. NbCXE Interacted with TMV CP

Plant resistance (R) genes confer resistance to various viruses. In some cases, viral CPs can be specifically recognized by the host R genes. For example, the Rx1 resistance gene of *Solanum tumberosum* can recognize the CP of *Potato virus X* (PVX) and induce a hypersensitive response that leads to resistance against PVX [42]. Moreover, it has been reported that the R genes in *Arabidopsis* and tomato conferred CP-mediated resistance to *Turnip crinkle virus* [43] and *Cucumber mosaic virus* [44], respectively. That the expression of TMV CP in plants confers resistance to infection by TMV and related tobamoviruses is because transgenic TMV CP in plants interferes with virus disassembly after virus entry [45]. Our study showed that there is interaction between host NbCXE and TMV CP. This provides a new perspective to the mechanism of CP-mediated resistance. The host carboxylesterase recognizes TMV CP through its direct interaction and link to the host defense signaling pathway to provide an enhanced resistance to virus infection. TMV CP plays an important role in TMV replication as it forms part of a viral replication complex [46]. The interaction between NbCXE and TMV CP has been shown in this study.

In conclusion, our study showed that NbCXE is a new plant resistance-related gene involved in host defense responses against TMV infection in *N. benthamiana* plants.

## Supplementary Materials

The following are available online at https://www.mdpi.com/1999-4915/12/2/195/s1, Table S1: The nucleotide sequences of all the primers used in this study.
