# Supplementary material for: A Conserved Carboxylesterase Inhibits Tobacco mosaic virus (TMV) Accumulation in Nicotiana benthamiana Plants
Source: Viruses. 2020 Feb 10;12(2):195. doi: 10.3390/v12020195 (PMC7077250; doi:10.3390/v12020195)
Supplement: Supplementary file 1 [file viruses-12-00195-s001.pdf]

Supplementary Table 1. The nucleotide sequences of all the primers used in this study.

Name	Nucleotide sequences (5'-3')
F-NbCXE-*Eco*RI	CCG**<u>GAATTC</u>** ATGGTGGCTG CTATTTCTTA TGATCCAA
R-NbCXE-*Bam*HI	CGC**<u>GGATCC</u>** TTAAACATCA TGGATAAAAT TCTTGAGATG
R-NbCXE$_{400}$-*Bam*HI	CGC**<u>GGATCC</u>** CGATAATTAA CTGACATAAT TACACAGCCA
NbCXE-F	ATGGTGGCTG CTATTTCTTA TGATCCAA
NbCXE-R	TTAAACATCA TGGATAAAAT TCTTGAGATG
NbCXE-qF	TCGGATACTT ACTGGCGA CTC
NbCXE-qR	CCACTTTAAT TCCAGCATCT G
TMV-R	TGGGCCCCTACCGGGGGTAA
TMV-qF	AGGTGTACAGGTACAATGCG
TMV-qR	ACGAGTAGCATCTAACGTTT
F-TMVCP-*Eco*RI	CCG**<u>GAATTC</u>** ATGTCTTACAGTATCACTAC
R-TMVCP-*Bam*HI	CGC**<u>GGATCC</u>** TCAAGTTGCAGGACCAGAGG
NPR1-qF	CGCCGGCGGAGATTACTTCACT
NPR1-qR	GGACTCCTCGCCGACAAAATG
HIN1-qF	TTCCGCCACCAGCAAAATC
HIN1-qR	TTAGGACGAAGAACGAGCCATA
HSR203J-qF	AGGCGGCGGCTTTTGTGTCA
HSR203J-qR	GAGAGGTCCCGGAGCCAGAGG
PR1-qF	GTG CCCAAAATTCTCAACA
PR1-qR	AAATCGCCACTTCCCTCAG
PR3-qF	TGGGGTTATTGCTGGCTTAG
PR3-qR	GGGTCATCCAAAACCAGAGA
Actin-qF	CTTGAAACAGCAAAGACCAGC
Actin-qR	GGAATCTCTCAGCACCAATGG

* The nucleotide sequences of enzyme digestion sites were bolded and underlined.
